# Proliferation in Minimal Invasive Samples of Canine Lymphomas: Ki67 Index in Previously Stained Cytology and Paired Cell Blocks

**DOI:** 10.3390/vetsci12060561

**Published:** 2025-06-08

**Authors:** Filipe Sampaio, Carla Marrinhas, Luísa Fonte-Oliveira, Ricardo Marcos, Pedro N. Oliveira, Marta Santos

**Affiliations:** 1 Cytology and Hematology Diagnostic Services, Laboratory of Histology and Embryology, Department of Microscopy, ICBAS–School of Medicine and Biomedical Sciences, University of Porto (U.Porto), Rua de Jorge Viterbo Ferreira, 228, 4050-313 Porto, Portugal; samp.filipe@gmail.com (F.S.); carla.marrinhas@euvg.pt (C.M.); luisa.oliveira@anicura.pt (L.F.-O.); rmarcos@icbas.up.pt (R.M.); 2 CEDIVET—Laboratório Clínico Veterinário, 4465-671 Leça do Balio, Portugal; 3 Hospital do Baixo Vouga, OneVet Group, 3750-742 Águeda, Portugal; 4 Anicura CHV Porto, 4100-320 Porto, Portugal; 5 Department of Population Studies, ICBAS, University of Porto, 4050-313 Porto, Portugal; pnoliveira@icbas.up.pt; 6 EPIUnit, ITR—Laboratory for Integrative and Translational Research in Population Health, University of Porto, 4050-600 Porto, Portugal; 7 Oncology Research, UMIB—Unit for Multidisciplinary Research in Biomedicine, ICBAS, University of Porto, 4050-313 Porto, Portugal

**Keywords:** canine lymphoma, Ki67, cell block, cytology, prognosis, immunocytochemistry, chemotherapy

## Abstract

Canine lymphoma (CL) is a heterogeneous neoplasm with varying prognoses, and Ki67 expression is a key marker for assessing tumor proliferation. This study aimed to compare Ki67 immunostaining in cytology smears (PSCS) and cell blocks (CBs) of canine lymphoma cases. Ki67 immunostaining was performed on 30 cases (26 nodal and 4 non-nodal) of CL, including B-cell, T-cell, and null-phenotype lymphomas. The Ki67 index was quantified manually using image analysis software as a support. The results showed Ki67 positivity in all CBs, with archival time affecting the antigenicity in PSCS, especially in samples older than two years. The Ki67 index in CBs of nodal CL were higher, and there was no significant agreement on Ki67 classification in PSCS and CBs. A univariate brief survival analysis was performed to preliminary evaluate the prognostic value of Ki67 in cytological samples. Ki67 indexes determined in cytology showed no significant association with survival. Cases of nodal CL with high Ki67 in CBs, if treated with chemotherapy, tended to survived longer (compared to those animals not treated with chemotherapy). These preliminary results showed that Ki67 immunostaining in CBs is more reliable for assessing CL proliferation and might offer predictive information. These findings highlight the potential of Ki67 quantification in CBs for supporting treatment decisions.

## Simple Summary

Canine lymphoma is a common and serious cancer in dogs and predicting how it will progress is important for choosing the best treatment. This study focused on a protein called Ki67, which indicates how quickly cancer cells are proliferating. Herein, Ki67 was quantified in two lymphoma samples: cytology smears and samples resulting from a processing of the lymphoma cells into a tissue-like form—called cell blocks. They found that cell blocks provided more reliable results. Although both methods showed similar levels of Ki67, only the Ki67 quantification in tissue-like samples predicted survival outcomes. Dogs with high Ki67 levels in cell blocks if treated with chemotherapy tended to have a survival advantage. This suggests that testing for Ki67 in cell blocks could help veterinarians to make better treatment decisions, potentially improving the chances of survival of dogs with lymphoma. More research is needed to confirm these findings, but this study represents an important step toward using minimal invasive samples for a prognostic assessment of canine lymphoma.

## 1. Introduction

Canine lymphoma (CL) is a heterogeneous group of neoplasms, with different clinical presentations. In dogs, the vast majority are non-Hodgkin lymphomas and the subtypes of CL are outlined in the World Health Organization (WHO) scheme, which considers, among others, the anatomical location, cellular morphology, tissue architecture, and immunophenotyping [1]. The histologic classification of CL cases is not always performed, considering the cost and the invasiveness of the biopsy procedure and the large turnover time, since it will delay the beginning of the chemotherapy regimen [2,3]. In a survey in the UK, 49 diplomats working in an oncology service mentioned that cytology was essential for diagnosing CL, and the majority confirmed that they performed histopathology only occasionally and that they did not consider this as an essential test [4]. Furthermore, 64% of 50 diplomats of clinical pathology gave a diagnosis of lymphoma without any further classification [4]. Several studies have been made in order to validate the use of complementary techniques, such as immunocytochemistry (ICC), molecular biology (PCR) and flow cytometry (FC) in CL samples using minimal invasive techniques, namely samples obtained by fine-needle aspiration (FNA) [3,5,6,7,8].

One of the relevant tumoral features that should be assessed during the workup of a CL case is the level of proliferation. Determining the growth fraction of lymphoma cells is essential to anticipate the behavior of the disease and response to treatment, thus allowing for the assessment of a tailored therapeutic protocol, specific to each case. One of the most used methods to determine tumor growth is the detection of Ki67 antigen by the monoclonal antibody MIB [6,7,9]. Ki67 is a nuclear protein that is expressed in all phases of the cell cycle (G1, S, G2 and mitosis), except for G0. Ki67 positive cells are counted to determine their index, and different cut-offs have been used to differentiate high- and low-grade lymphomas by FC [6,7,9,10] and by immunohistochemistry (IHC) [11] or by FC and IHC [12]. A general agreement between IHC and FC has been determined, but with a wide range of confidence intervals [12]. Some cases showed discrepancies between the two quantification methods, mainly due to differences in the selection and number of the cells counted. For determining the Ki67 index in IHC, the pathologist subjectively selects the most proliferative areas in the slide and usually considers 500 cells for computing the index. In contrast, a larger number of cells in suspension are counted by FC, without any previous subjective selection [12].

Typically, cases with low Ki67 indexes are associated with a longer survival time. In canine diffuse large B-cell lymphoma (DLBCL), Ki67 values over 107 positive cells per 5 high-power fields were associated with shorter survival when treated with CHOP chemotherapy [13]. On the other hand, the prognostic role of this Ki67 index in rare types of lymphoma, such as small cell B-cell subtypes (SCBCL), remains to be elucidated. Recent findings indicated that SCBCL with high Ki67 expression behave aggressively, challenging the typical association of small cell phenotype with a more indolent form of disease [9]. However, contradictory results have been published, with one study showing that an intermediate Ki67 index was associated with better prognosis, compared with low or high Ki67 indexes [7]. More recently, a study found that DLBCL harboring mutations in the POT1 gene had significantly higher Ki67 indexes compared to those with wild-type POT1 [14].

Some evidence exists that Ki67 index can be computed in FNA smears using ICC and the monoclonal antibody MIB [5]. Still, it is well acknowledged that the suitability of unstained cytologic slides for performing ICC is highly dependent on its cell representativeness and preservation, both unknown before staining the FNA smear [3]. By choosing previously stained cytology smears (PSCS) for ICC, this issue is circumvented. Immunophenotyping using ICC and PSCS is currently offered by many commercial laboratories, even if inconclusive results may be obtained in some cases [3]. It has been highlighted that in an ideal scenario, the ICC in PSCS should be complemented by IHC on cell blocks (CBs), obtained from needle rinses [3]. This approach would guarantee that, in any inconclusive results in PSCS, the same marker or others could be applied on the CBs. The suitability of PSCS for assessing and counting Ki67 positive cells is still unknown. A comparison of ICC for Ki67 in PSCS and IHC on CBs obtained from needle rinses has never been performed, to the best of our knowledge.

Considering this, we aimed to (1) study the influence of the archival time of the FNA smears and CBs of CL on the Ki67 antigenicity; (2) evaluate and compare the Ki67 index obtained by ICC in PSCS and matched CBs; (3) assess the association between Ki67 index classification in PSCS and CBs and survival time and evaluate survival differences within the high Ki67 index group in relation to treatment.

## 2. Materials and Methods

### 2.1. Sample Selection and Immunostaining for Ki67

Archived non-Hodgkin nodal CL cases were retrospectively selected from the Cytology Diagnostic Services, ICBAS, University of Porto and a private veterinary hospital (Hospital do Baixo Vouga, OneVet Group). The collection of samples was performed by the reference clinician for diagnostic purposes, with informed consent provided by the owners for the use of the biological and clinical data for research purposes. Additionally, cases of CL affecting other organs were also included to diversify the sample. In all the cases, two cytopathologists (FS and MS) reviewed the FNA slides, and cases were enrolled only if (1) one representative cytology slide with good cell preservation was available; (2) a CB from the matched case had been obtained; (3) the immunophenotype had been determined by IHC on the CB. Cases with poor cellularity or inadequate cell preservation in cytology were excluded. The CBs were prepared using the cell-tube block technique as previously described [15]. PSCS had been Romanowsky-stained and were scanned using an Olympus VS110 slide scanner (Olympus, Japan) for archival and reviewing purposes. The corresponding CBs were routinely sectioned and 4 µm sections used for IHC. Therefore, each case consisted of a PSCS and a matched CB slide, which were processed together in the same set of immunostaining. The ICC protocol was optimized based on various published studies [3,8,16]. Positive controls obtained from normal canine lymph nodes (collected during necropsy) were included in all immunostaining runs.

For ICC using PSCS, the slides were immersed in xylene until the coverslip detached and left in xylene until the remaining mounting medium was completely removed. They were then transferred to 100% alcohol for 5 min, followed by 95% alcohol for another 5 min. Subsequently, slides were immersed in cold acetone for 15 min and air-dried. Antigen retrieval was performed using TRIS-EDTA (pH = 9.0) with two heating cycles: the first for 3 min at 750 W and the second for 7 min at 160 W, as previously reported [16]. For IHC using CB, the slides were deparaffinized in xylene for 10 min and then immersed in fresh xylene for an additional 10 min. The slides were transferred to 100% alcohol for 5 min, followed by two sequential immersions in 95% alcohol for 5 min each. They were then placed in 70% alcohol for 5 min and finally washed in tap water for 5 min. Antigen retrieval was performed using a citrate buffer (pH 6.0) at 95 °C for 3 min in a pressurized cooker, followed by cooling to room temperature. From this point onward, both PSCS and CB slides were simultaneously processed using the same protocol. A polymer-based immunostaining kit was used (Novolink Max Polymer Detection System, Leica Biosystems Newcastle, Newcastle, UK). The primary anti-Ki67 antibody (clone MIB1, Dako, Glostrup, Denmark) was applied at a 1:100 dilution in phosphate-buffered saline (PBS) with 5% bovine serum albumin (BSA) for 2 h at room temperature in a humidified chamber. For negative controls, the primary antibody was replaced with PBS containing 5% BSA. Endogenous peroxidase activity was blocked by incubating the slides for 10 min with 3% hydrogen peroxide. The final reaction was visualized using the chromogen 3,3′-diaminobenzidine (DAB, Novolink Max Polymer Detection System, Leica Biosystems Newcastle, Newcastle, UK), and nuclear counterstaining was performed with hematoxylin.

### 2.2. Quantification of the Ki67 Index

All slides were evaluated by a single observer (FS). To assess the Ki67 index, cells were counted in 10 fields at 400× magnification. The Ki67 index was calculated as the number of Ki67-positive cells divided by the total number of lymphoid cells in each field. The image analysis QuPath software (version 0.4.3) [17] was used to assist the manual counting of the nuclei in PSCS and in CB samples. Only cells with clear nuclear Ki67 staining were considered positive. Any dubious or nonspecific staining, as well as ambiguous or unclear nuclear structures, were excluded. To avoid eventual bias, the observer (FS) was blinded to the identification of cases.

In order to classify the proliferation of CL cases, two previously reported thresholds were used. The Ki67 index was classified as low or high according to the cut-off (12.2%), which has been defined by FC [6]. Additionally, the three-tier classification of low (≤20% Ki67+ cells), moderate (20–40% Ki67+ cells), or high index (>40% Ki67+ cells) was also applied [6].

### 2.3. Follow-Up Data

The follow-up data was retrieved from the medical records by the reference clinician. The therapeutic regimen for each animal was documented, with particular emphasis given to the treatment protocol (specifically whether the animal received chemotherapy or not). Disease-specific survival was defined as the period between the cytologic diagnosis of lymphoma and the death (or euthanasia) due to disease progression and/or loss of quality of life due to the neoplastic disease.

### 2.4. Statistical Analysis

Statistical analysis was performed in IBM SPSS Statistics for Windows, version 28.0 (IBM Corp, Armonk, NY, USA). The raw values and arcsin-transformed values of the Ki67 index in PSCS and CB were compared using the T-test for paired samples and the Pearson Chi-squared test. The agreement between Ki67 index classifications in PSCS and CBs was visualized in a double entry table and calculated using the Cohen’s kappa (k) statistics. The following interpretation of k was considered: ≤0 = poor agreement, 0.01–0.20 = slight agreement, 0.21–0.40 = fair agreement, 0.41–0.60 = moderate agreement, 0.61–0.80 = substantial agreement, and 0.81–0.99 = almost perfect agreement. The difference in the Ki67 index in PSCS and CBs according to the archival time was represented in Bland–Altman scatter plots. The Kaplan–Meier method and log-rank tests were used to estimate and compare the survival of dogs with CL of different Ki67 index classification categories. *p* values ≤ 0.05 (two-tailed) were considered statistically significant.

## 3. Results

### 3.1. Clinicopathological Features and Ki67 Antigenicity in Cytology and Cell Blocks

A total of 30 cases were included in this study, comprising 19 B-cell lymphomas, 9 T-cell lymphomas, and 2 null-lymphomas (i.e., non-B-non-T). Most were nodal lymphomas (26 cases: 17 B-cell, 7 T-cell, and 2 null-lymphomas), but alimentary (2 cases: 1 enteropathy-associated large T-cell lymphoma and 1 diffuse large B-cell lymphoma, by histopathology), cutaneous (1 large T-cell lymphoma, by histopathology), and splenic (1 medium B-cell lymphoma) were also included. All the cases were composed by medium (nucleus size of 2–2.5 red blood cells) to large cells (nucleus size larger than three red blood cells). The cutaneous and intestinal lymphomas were large-cell subtypes. The cohort consisted of 12 females and 14 males, with a mean age of 7.13 years (range: 1–15 years) (age information was not available in seven cases). The cases had been obtained from an academic laboratory (18) and private hospital (12); in these latter cases, the corresponding PSCS slides were not coverslipped. The archival time of the cases was less than a year in 13 cases. Follow-up data was available for 26 cases: among them, 8 cases were treated with a multi-agent chemotherapy protocol, while 18 received corticosteroid therapy alone. The mean and median overall survival times of the cohort were 76.5 days and 51 days, respectively.

**Figure 1:**
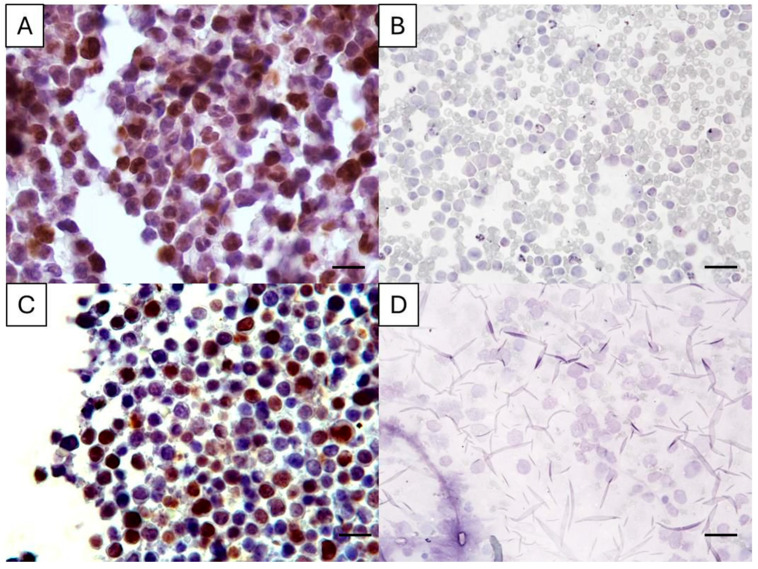
Ki67 immunostaining in cell-tube block slides (**A**,**C**) and the corresponding previously stained cytology smear (**B**,**D**) of two canine lymphoma cases. In A, the Ki67 index was 30%, and in C it was 54%, while in the matched cytology slides the Ki67 antigenicity was abolished. Magnification: 400×, bar = 35 µm. Diaminobenzidine chromogen, hematoxylin counterstain.

The Ki67 antigenicity could not be detected in two PSCS. These two cases had an archival time > 2 years and, despite no Ki67-positive cell having been observed in FNA smears, the corresponding CBs had a Ki67 positivity of 30% and 54% (Figure 1). In all CB slides, Ki67-positive cells were detected; however, in two cases, the Ki67 index was markedly lower (1% and 4%) compared to the corresponding PSCS (45% and 60%, respectively).

### 3.2. Ki67 Index Determination and Classification

During the quantification of Ki67 positive cells, a mean of 2997 (±2130) and 3534 (±3660) total cells were counted in PSCS and CBs, respectively. The Ki67 index in PSCS was lower compared to the index computed in the corresponding CB in 18 cases. The mean and median of the Ki67 index in PSCS were 32.8% (±22.7%) and 30.4%, respectively, while in CB, the mean and median of the Ki67 index were 42.0% (±43.4%) and 43.4%. The paired samples correlation between the Ki67 indexes in cytology and CBs was weak and non-significant. When considering only nodal CL (n = 26), the mean and median of the Ki67 index in PSCS were 33.6% (±23.5%) and 31.6%, respectively, while in CBs, the mean and median of the Ki67 index were 49.9% (±20.4%) and 46.3%. These differences were statistically significant (*p* = 0.003). Concordantly, the paired samples correlation between the Ki67 indexes in cytology and CBs was also weak and also non-significant.

**Figure 2:**
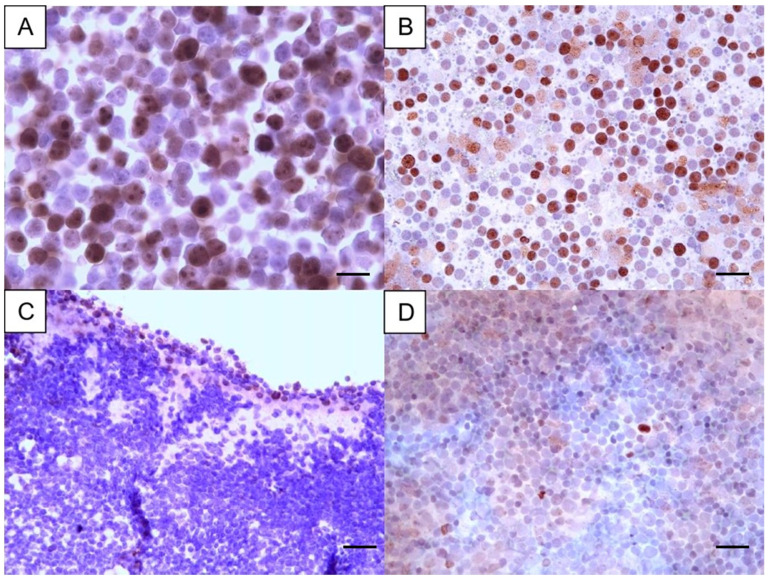
Ki67 immunostaining in two cases of canine lymphoma, with each case represented by a cell-tube block and the corresponding cytology smear. The case shown in images (**A**,**B**) was classified as high, with a high proliferative index—Ki67 positivity of 66% in the cell-tube block and 69% in the cytology smear. In contrast, the case depicted in images (**C**,**D**) was categorized as low, with a Ki67 positivity of 10% in the cell-tube block and 7% in the cytology smear. Magnification A: 1000× (bar = 23 µm). Magnification B, C and D: 400× (bar = 35 µm). Diaminobenzidine chromogen, hematoxylin counterstain.

Using the cut-off of 12.2%, 6 cases were low-proliferative, and 24 cases were highly proliferative in PSCS, while in CBs, 4 were low and 26 highly proliferative (Table 1; Figure 2). The agreement between PSCS and CB classification was obtained in 24 cases (2 low and 22 high). Despite this, the kappa value was small, and the agreement was non-significant, when all cases were considered. In order to disclose if the agreement might be dependent on the archival time of the cases, the cases with an archival time of less than 12 months (n = 13) were considered separately. In those, the mean Ki67 positivity was 36.6% (±22.5%) in PSCS, being 49.6% (±16.3%). The kappa value was higher (k = 0.46, *p* = 0.133), but with no statistical significance. For cases with longer archival time (≥1 year), the mean Ki67 index in PSCS was 30.3% (±23.1%), and 36.9% (±25.4%) in CBs. No correlation existed and these differences were non-significant. When considering nodal lymphomas (n = 26), 6 cases were also low proliferative, and 20 cases were high proliferative in PSCS, while in CBs, only 1 was low and 25 were highly proliferative. Similarly, the agreement between PSCS and CB Ki67 index classification was non-significant in nodal CL.

The Ki67 index thresholds: ≤ 20%, >20%/≤40% and >40% were also used to perform a three-tier classification (low, intermediate, and high) of the proliferation status of all the CL and for the nodal CL subgroup. As a result, 11, 8, and 11 cases were classified as low, intermediate, and highly proliferative, respectively, in PSCS (Table 2). These figures changed in CBs, since 5, 8, and 17 cases were classified as a low, intermediate, and highly proliferative, respectively. Of the 17 cases with high Ki67 index in CBs, 9 cases had lower Ki67 indexes in the corresponding PSCS (Table 2). Accordingly, the agreement of the three-tier classification performed in the two specimens was fair k = 0.24, *p* = 0.045). In nodal lymphomas (n = 26), the agreement of the 3-tier classification system of Ki67 in PSCS and CBs was slightly better, despite still being fair (k = 0.35; *p* = 0.003). The cut-off of 20% was used to reclassified the lymphomas cases, with the intermediate and high categories being grouped as “new” high (Table 3).However, this reclassification did not improve the agreement between Ki67 index in PSCS and CBs, with very low kappa values (k < 0.2).

### 3.3. Preliminary Assessment of the Prognostic Significance of Ki67 Index Classification

Follow-up data was available for 26 animals, including information about their therapeutic regimen. The mean and median disease-specific survival time in this cohort, independently of the therapy instituted, was 76 and 51 days, respectively. For animals surviving ≤ 51 days (n = 13), the mean Ki67 index was 32.2% ± 23.5% and 39.5% ± 25.9% in PSCS and CBs, respectively. In animals surviving ≥ 52 days (n = 13), the Ki67 index in cytology was, on average, very similar to that computed in animals with lower survival, while in CBs the Ki67 index mean was higher in long survivors (50.1% ± 15.8%). It should be stressed that these values are raw and do not consider if the animals received chemotherapy or not.

An initial survival analysis was conducted considering all the CL cases and the Ki67 index classifications. Kaplan–Meier survival curves were generated to represent the survival time of the CL categorized as low and high (using the cut-off of 12.2%) according to the Ki67 index in PSCS and in CBs. Log-rank test revealed no statistically significant differences in survival of dogs with low and highly proliferative lymphomas. Similarly, when the cases were classified as low, intermediate, and highly proliferative both in PSCS and CBs, the differences in survival curves were not statistically significant. In both Ki-67 index classifications systems, the number of CL classified as low in CBs was very few: 4 cases in the two-tier classification (cut-off 12.2%) and 5 cases in the three-tier classification (cut-offs 20% and 40%). Considering that, in the following steps of the statistical analysis, only the data of cases classified as having a high Ki67 index (i.e., cases with >12.2%) were included. In the chemotherapy-treated group, the median and mean survival times were 121 days and 141 days (SE: 40.6; 95% CI: 61–221), respectively, while the group non-treated with chemotherapy had a median survival time of 42 days and a mean survival time of 62 days (SE: 16.1; 95% CI: 30–94). The log-rank test indicated a trend towards improved survival in cases with high Ki67 index in cytology, when chemotherapy was instituted (*p* = 0.059). In cases with a high Ki67 index computed in CB, the survival curves of those treated with chemotherapy (n = 7) and those not subjected to chemotherapy (n = 16) were statistically different (*p* = 0.036).

**Figure 3:**
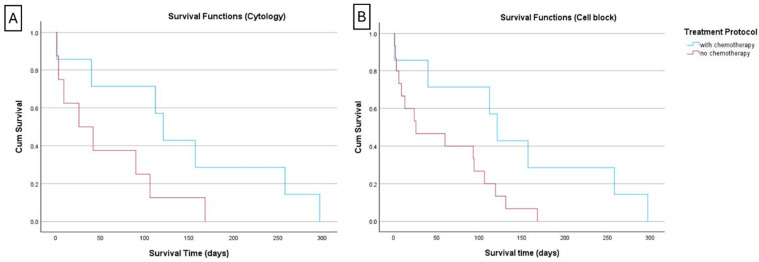
Kaplan–Meier curve of canine lymphoma cases with a high Ki67 index > 20% (reclassification of the “intermediate + high” categories in a new high category). (**A**) When the reclassification was performed using the cytology Ki67 values, there was a trend toward improved survival in chemotherapy-treated animals compared to those dogs not treated, but the difference did not reach a statistical significance (*p* = 0.077); (**B**) when the same was performed using the Ki67 indexes computed in the cell-tube blocks, the survival was significantly improved when the cases with Ki67 index > 20% were treated with chemotherapy (*p* = 0.040).

Further Kaplan–Meier curves were generated to compare survival between cases reclassified as “low” (i.e., ≤20%) versus cases “intermediate + high” (i.e., >20%). For the cytological classification of Ki67, no significant difference in survival was observed between the “low” and “intermediate + high” categories. The survival analysis was also performed with this stratification to the treatment protocol (Figure 3). The “intermediate + high” group of chemotherapy-treated animals (n = 7) had a median and mean survival time of 121 and 141 days (SE = 41; 95% CI: 61–220), respectively. These figures tended to be higher than those of the non-treated subgroup (n = 8) [26 and 56 days (SE = 21; 95% CI: 14–97)], but this difference did not reach a statistical significance (*p* = 0.077). For Ki67 index classification in CB, it was not possible to compare survival between animals classified as “low” (i.e., ≤20%) versus “intermediate + high” (i.e., >20%) because only two cases were classified as low (Table 3). By comparing the survival curves of animals classified as “intermediate + high” and treated with chemotherapy *versus* those that were not treated, it was clear that the former had a significant longer survival time (Figure 3) (chi-square value of 4.231, *p* = 0.040). Indeed, in the chemotherapy-treated group (n = 7), the median survival time was 121 days, with a mean survival time of 141 days (SE = 41; 95% CI: 6–220). In the non-treated group (n = 15), the median survival time was 26 days, and the mean survival time was 57 days (SE = 15; 95% CI: 28–86).

By focusing on the survival analysis of the nodal large-cell lymphomas, animals with CL classified as highly proliferative in CBs (Ki67 > 12.2%) and treated with chemotherapy survived on average 124 days (median 57 days), while animals not treated with chemotherapy had 67 and 40 days of mean and median survival times, respectively. Yet, the difference was not statistically significant, but it should be stressed that the sample was limited to 20 cases. A similar result (longer survival times in treated animals, but the difference did not reach the statistical significance) was achieved when the classification of the proliferation in CB was carried out using the Ki67 index cut-off of 20% [“low” (i.e., ≤20%) versus “intermediate + high” (i.e., >20%)] of positive cells.

## 4. Discussion

The Ki67 is an important prognostic marker in different canine neoplasia including CL, with higher expression levels typically linked to poorer survival outcomes [13]. The quantification of the Ki67 lymphoma positive cells is usually performed in biopsy samples using IHC or by using FC, which requires cell suspensions and is much more expensive [6,7,9,10]. Cytology is usually the first diagnostic test performed in cases of peripherical lymphoma (e.g., nodal or cutaneous) and lymphomas in anatomical areas accessible by ultrasound-guided fine-needle aspiration [4]. Although the architectural patterns and the full grading of CL cannot be established by cytology [1], the cytological samples are easy to obtain, minimally invasive, and suitable for a rapid on-site evaluation [15]. Thus, efforts have been made to complement the cytological diagnosis with prognostic data, thus supporting the clinical and informed tutor co-decision [15]. Following this approach, a comparison of Ki67 immunostaining in PSCS and paired CBs was performed herein, filling the gap regarding the suitability of those samples to establish the Ki67 indexes in CL. It was demonstrated that Ki67 positive cells can be detected in PSCS, as well as in CBs, with different archival times. However, there was a complete loss of antigenicity in a few cases of PSCS, and IHC issues were also noted in CBs. Inconsistent results in PSCS have already been reported when lymphoid markers were used for immunophenotyping purposes [3,18]. It is important to stress that we decided to perform the Ki67 immunostaining in a single PSCS and single CB section. A different result might have been obtained if the immunostaining was repeated in another smear or section. In this regard, the CB is advantageous, as several sections can be easily obtained (Figure 1, Supplementary Materials). In the two CL cases for which the first IHC staining in CB presented few positive cells, another CB section was immunostained and a higher positivity was observed. We hypothesized that an analytical factor (e.g., drying of the slide, low volume of one solution over the section during a step of the protocol) was the cause of a very low positivity after the first IHC.

Previous studies have reported that the Ki67 expression of FNA samples fixed with 10% neutral buffered formalin or 96% alcohol declined over time [19] and this was why we separately evaluated samples with more and less than one year. The agreement between the classification of the Ki67 index as low/high in PSCS and CB improved in more recent samples, but, still, no statistical significance was attained. This was probably due to the relatively small sample size of this cases series. However, our results suggest that recent smears are more suited for Ki67 immunostaining, and this deserves further studies.

In our study, most of the included cases were nodal lymphomas with a multicentric presentation, which is the most frequent clinical presentation of non-Hodgkin lymphomas in dogs [1]. A small subset of non-nodal lymphomas (n = 4) was also included in order to better represent the diversity of the anatomic presentation of non-Hodgkin CL. Considering that the biological behavior of CL is dependent on the clinical and anatomical presentation, the statistical analysis was performed initially considering all the cases and then focusing on the nodal CL.

In our case series, the CL with low proliferation were underrepresented [4 out of 26 cases (15%)]. Such a low percentage of CL with low Ki67 index has been described in previous studies (10–23% of all cases) using other techniques and specimens. Still, this limited the statistical analysis in the highly proliferative CL [6,7]. Dogs with high Ki67 index in CBs who received chemotherapy had a median survival time of 121 days, which is comparable to other reports, and in the range of 63-day median survival reported for a 19-week CHOP-based protocol [13] and 173 days of average survival time of animals treated by a modified Wisconsin–Madison protocol [7]. Despite this, the comparison of the survival times between studies should be made with caution, considering the typical clinicopathological heterogeneity of CL. Indeed, it is always necessary to consider, for example, the impact of both lymphoma subtype and treatment protocol on survival outcomes [9], and multivariate regression analysis are needed. Herein, the small sample size of this cohort jeopardized the analysis of other explanatory variables (rather than Ki67). Moreover, we included a mixed population of dogs without clinical staging, cases were not-otherwise-classified lymphomas, and we grouped together B- and T-cell lymphomas, which are known to have a different prognosis. For the sake of clarification, the results of our survival study should be viewed as part of the clinical validation of Ki67 index determination in minimal invasive samples and as a preceding step for more detailed survival validation studies.

In order to retrieve prognostic information from the Ki67 index, it is important to define their thresholds. Considering the present findings, the 12.2% threshold in CBs seems to be more advantageous for prognostic definition. A high Ki67 index in CBs seems to be important to prognostication, since those animals treated with chemotherapy presented a significant longer survival. This index in CBs is especially useful for clinical settings where FC is not available and a rapid result is needed. Considering that FNA needle rinses fluids can be obtained at the first presentation of a suspected case of CL and the CB processing and subsequent IHC results take about 3 working days, such a quantification in CBs would support the therapeutic decision and the prognosis definition. Still, it must be kept in mind that cytology smears, CBs, and FC provide a different number of cells and may not reflect the distribution of cells seen in tissues (i.e., these may be biased samples of the tissue). Therefore, the refinement of thresholds is needed for these types of platforms for immunostaining (as has occurred for FC samples [6,7]). In this vein, future studies should include not only PSCS, but also the corresponding CB and, ideally, lymph node biopsies.

## 5. Conclusions

In conclusion, the marker Ki67 can be detected by immunolabelling in PSCS and CBs of CL with different archival times, despite the fact that a loss of antigenicity might occur in archived PSCS. There was no agreement on the classification of a Ki67 index computed in PSCS and paired CBs. Still, the institution of chemotherapy in dogs with a CL with a high Ki67 index computed in CBs tended to give them a survival benefit. This finding pointed to a possible clinical value of the Ki67 classification in CBs by adding information to support the treatment decision in individual cases of CL. Herein, the utility of CBs for ancillary techniques was substantiated: in addition to being a good platform for immunophenotyping CL [3,15,18,20,21], it is useful for assessing and quantifying the cell proliferation. So, in order to provide a better service to the animal and to their owner, it is advisable that the veterinary practitioner, when dealing in a suspected case of CL, make efforts to perform a needle rinse and obtaining a CB.

## Figures and Tables

**Table 1:** Contingency table comparing two-tier classification of the Ki67 index using the threshold 12.2% (low < 12.2% and high ≥ 12.2%) in previously stained cytology smears and corresponding cell-tube blocks of canine lymphomas.

		**Cell block**		
**Cytology**		Low	High	Total
	Low	2	4	6
	High	2	22	24
	Total	4	26	30

**Table 2:** Contingency table comparing a three-tier classification of the Ki67 index using the thresholds 20% and 40% [low (≤20%), intermediate (>20% and ≤40%), or high (>40%)] in previously stained cytology smears and corresponding cell-tube blocks of canine lymphomas.

		**Cell block**			
**Cytology**		Low	Intermediate	High	Total
	Low	2	2	7	11
	Intermediate	1	5	2	8
	High	2	1	8	11
	Total	5	8	17	30

**Table 3:** Contingency table comparing a reclassification of the Ki67 index, considering the low category (Ki67 index ≤ 20%) and a new high category (Ki67 index > 20%) in previously stained cytology smears and corresponding cell-tube blocks of canine lymphomas.

		**Cell block**		
**Cytology**		Low	High	Total
	Low	2	9	11
	High	3	16	19
	Total	5	25	30

## Data Availability

All data generated or analyzed during this study are included in this published article and its supplementary information files.

## Supplementary Materials

The following supporting information can be downloaded at: https://www.mdpi.com/article/10.3390/vetsci12060561/s1, Figure S1: Ki67 immunostaining in two cases (A, B, C and D, E, F) of canine lymphoma.
