# Supplementary material for: Proliferation in Minimal Invasive Samples of Canine Lymphomas: Ki67 Index in Previously Stained Cytology and Paired Cell Blocks
Source: Vet Sci. 2025 Jun 8;12(6):561. doi: 10.3390/vetsci12060561 (PMC12197597; doi:10.3390/vetsci12060561)
Supplement: Supplementary file 1 [file vetsci-12-00561-s001.zip › vetsci-3585262-supplementary.pdf]

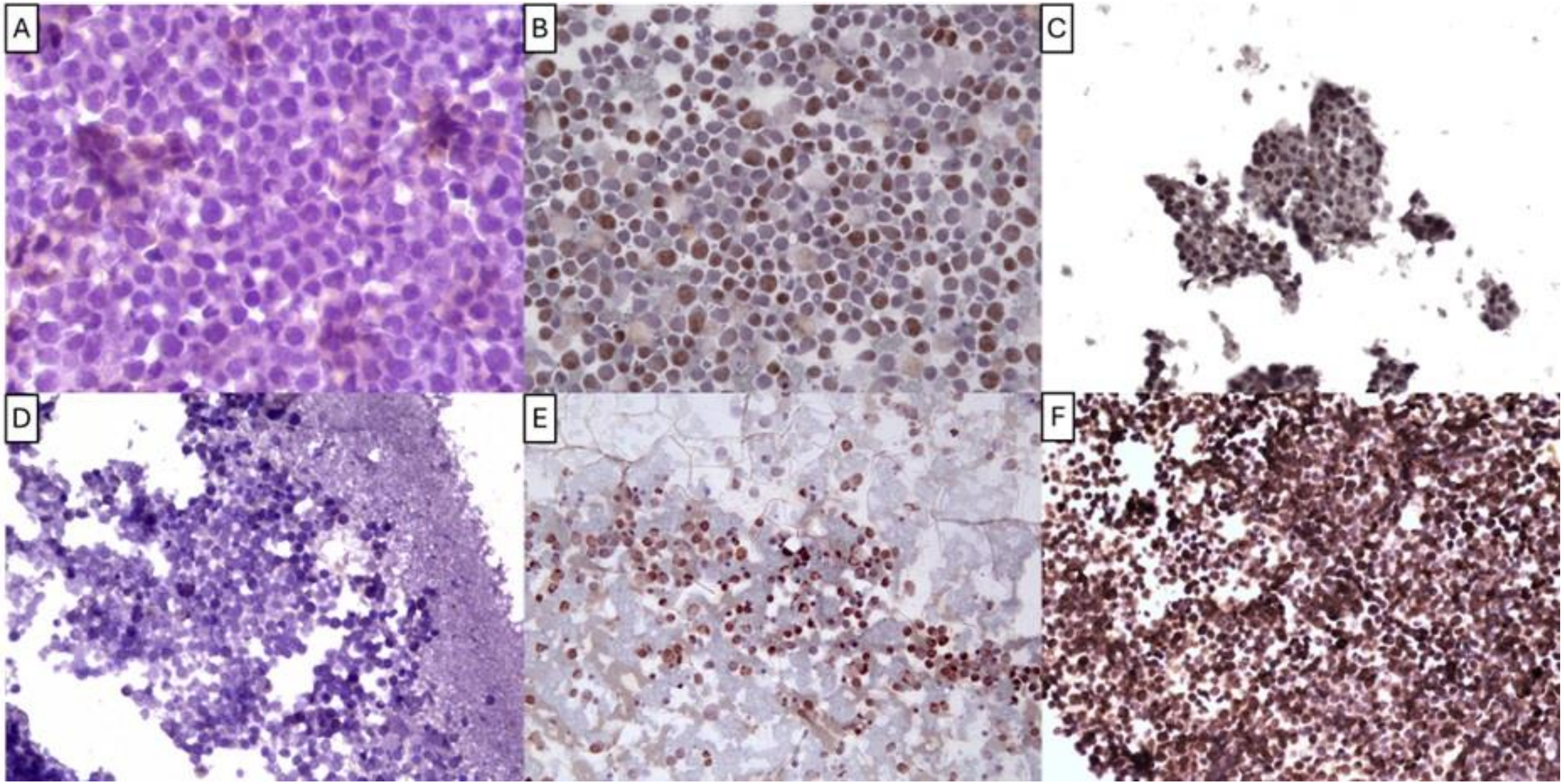

Figure S1 supplemental. Ki67 immunostaining in two cases (A, B, C and D, E, F) of canine lymphoma. A and D corresponding to cell tube blocks and B and E are the corresponding cytology smear, respectively. In the first immunohistochemistry in cell tube block slides a very low Ki67 positivity was obtained compared to the immunolabelling in the cytology. The immunohistochemistry was repeated in successive cell tube block section and the Ki67 index was markedly increased (C and F). Magnification: 400×. Diaminobenzidine chromogen, hematoxylin counterstain.
